# Predictors of tuberculosis knowledge, attitudes and practices in urban slums in Nigeria: a cross-sectional study

**DOI:** 10.11604/pamj.2019.32.60.14622

**Published:** 2019-02-04

**Authors:** Mobolanle Rasheedat Balogun, Adekemi Oluwayemisi Sekoni, Seema Thakore Meloni, Oluwakemi Ololade Odukoya, Adebayo Temitayo Onajole, Olukemi Arinola Longe-Peters, Folasade Tolulope Ogunsola, Phyllis Jean Kanki

**Affiliations:** 1 Department of Community Health and Primary Care, College of Medicine of the University of Lagos, Lagos, Nigeria; 2 Department of Immunology and Infectious Diseases, Harvard TH Chan School of Public Health, Boston, MA, USA; 3 Department of Community Health, Lagos University Teaching Hospital, Lagos, Nigeria; 4 Department of Medical Microbiology and Parasitology, College of Medicine of the University of Lagos, Lagos, Nigeria

**Keywords:** Cross-sectional study, Nigeria, predictors of TB KAP, tuberculosis, vulnerable communities

## Abstract

**Introduction:**

Nigeria is among six countries responsible for the majority of tuberculosis (TB) cases in the world. The Nigerian government has emphasized community-based case finding to increase detection of TB. This process requires efforts to improve knowledge, attitudes and practices (KAP) of TB, particularly in the poorest of communities. This study presents data from a KAP survey administered in two underserved Nigerian communities.

**Methods:**

a structured survey was administered by trained interviewers among adult residents in two slum communities in Lagos, Nigeria. Participants were selected through multistage random sampling. KAP scores were computed and the predictors of higher scores were assessed.

**Results:**

a total of 504 respondents were surveyed. The mean KAP scores were relatively low: 9.8 ± 7.1 for knowledge (out of a maximum 34), 5.3 ± 3.4 for attitude (maximum = 10), and 5.2 ± 1.5 for practice (maximum = 7). The predictors of good knowledge were increasing age, post secondary education and professional occupation. The predictors of positive attitude were post secondary education and good TB knowledge. Good knowledge was a predictor of good practice

**Conclusion:**

our findings underscore the need to improve the education about TB in underserved communities. Improving KAP scores will ultimately lead to higher rates of TB detection and treatment.

## Introduction

Tuberculosis (TB) is one of the top 10 causes of death worldwide with an estimated 10.4 million new cases and 1.8 million deaths from the disease in 2015; over 95% of these deaths occurring in low-and middle-income countries (LMIC) [1]. In the era of the Sustainable Development Goals, the World Health Organization (WHO) produced “The End TB Strategy” with the aim of a 90% reduction in TB deaths and 80% reduction in TB incidence rate by 2030, compared with 2015 [2, 3]. Africa is home to 16 of the 30 highest TB burden countries in the world [4]. This high burden can be, in part, attributed to issues associated with population growth, the HIV/AIDS epidemic, multi-drug resistant TB, inadequate health systems and poverty [5]. In Nigeria, TB diagnosis and treatment is free under the Stop TB initiative [6]. Despite existence of the program, the nation is among six countries responsible for 60% of the total TB burden in the world with an incidence rate of 322 per 100 000 population in 2015 [1, 2]. The strategies to control TB in the country include: expansion of the directly observed treatment, short-course (DOTS) program; TB/HIV collaborative activities; multi-drug resistant (MDR) TB services; engagement of all care providers; engagement of communities and patients in TB activities; and, advocacy, communication and social mobilization (ACSM) to improve community awareness on TB [6]. To achieve significant progress in the control efforts, community members need to be knowledgeable about TB and have positive attitudes and satisfactory preventive practices. DOTS relies on suspect TB cases presenting themselves for care at health facilities, which can be facilitated when community knowledge is high and stigmatizing attitudes are low [7]. Poor communities are more vulnerable to TB because of lack of awareness, overcrowded and substandard living conditions, poor nutrition, intercurrent disease, and economic, geographical, social and cultural barriers to accessing TB services [8, 9]. To address these issues, the Nigerian government has emphasized community-based case finding with a goal of increasing detection of presumptive TB cases referred by community volunteers between 2013 and 2014 from 11% to 23% [6]. As passive case finding remains the mainstay in routine control programs, improving community knowledge and attitudes, from what was previously determined to be low levels, will be essential in this community-based case finding process [7, 10]. To examine the current level of knowledge and attitude in the “End TB Strategy” era with the goal of guiding future interventions, we evaluated the knowledge, attitudes and practices in two vulnerable communities in Nigeria.

## Methods

**Study areas:** this cross-sectional study was carried out in the Idi-Araba and Okokomaiko communities, which fall under the Mushin and Ojo (urban), respectively, local government areas (LGAs) in Lagos, Nigeria. Both communities are large, congested slums with poor sanitation and low-quality housing. The study population was comprised of adults living in either of the communities.

**Sampling:** a calculated sample size of 504 respondents (252 in each community) were selected by a multi-stage sampling technique. A list of streets was obtained from the Community Development Association (CDA) of each community. Sixteen streets were selected from each community by simple random sampling and the habitable houses on each street were enumerated. Systematic random sampling was then used to select houses on the streets, while selection of households in the houses was chosen by simple random sampling (in instances where there were multiple households per house, one household was selected from each house). One eligible respondent was then selected per household by simple random sampling (where there was more than one). If the occupants of the house were unavailable, or if eligible household members were not willing to participate in the study, the next consecutive house was used.

**Data collection methods:** data were collected using a structured interviewer-administered questionnaire, which was adapted from the WHO sample ACSM KAP survey questionnaire [11]. The tool was pre-tested in a similar community outside of the study sites and appropriate amendments were made. The survey collected information on personal characteristics, TB knowledge and awareness, TB attitudes and care-seeking behavior, TB attitudes and stigma and TB information and preventive practices. Face-to-face interviews were conducted by six trained research assistants with post-secondary education and fluent in the local languages. The research assistants were supervised by some members of the research team. Interviews were conducted in the homes of respondents and took between 15 to 25 minutes to complete.

**Data analysis:** data were analyzed using Statistical Package for Social Sciences (SPSS) version 15 (SPSS Inc, Chicago, IL). The overall KAP of respondents were assessed. For knowledge, 11 questions were scored and some allowed multiple responses; a score of one was given to correct responses and the possible scores ranged from 0-34. Ten questions on attitude were scored and the positive responses were each given a score of one. For preventive practices, 4 questions were scored, 3 of which were on a three-point scale graded 0-2; the maximum obtainable score was 7. The cut-off for what was considered to be “good” knowledge, “positive” attitude and “good” practice were the mean values for each of these, respectively. Statistical differences within interview sites for categorical variables were evaluated using the Chi-squared or Fisher's exact tests, as relevant and for continuous variables using the Wilcoxon rank sum test. Bivariate associations between sociodemographic variables and KAP levels were evaluated using Chi-squared test. Logistic regression was conducted to examine multivariate associations between respondents' characteristics and TB knowledge, attitude and practices. Variables with statistical differences at p<0.2 in bivariate analyses were included in logistic regression analyses using a block entry approach. Odds ratios (OR) and 95% confidence intervals (CI) were computed for each predictor variable. Level of significance was set at 0.05.

**Ethical Approval:** the study proposal was approved by the Health Research and Ethics Committee of Lagos University Teaching Hospital. Written informed consent was obtained from the respondents prior to the administration of questionnaires and confidentiality was maintained by not using identifiers.

## Results

**Socio-demographic characteristics:** the respondents ranged in age from 18 to 89 years with a median age of 34 years (IQR: 27-43). Most respondents were female (60.7%), married (65.5%), had at least a secondary-level education (62.5%) and were employed (78.0%). Over a third (45%) of them were semi-skilled. The two communities differed in ethnicity, employment status and occupation, with Idi-Araba having higher proportions of unemployed and unskilled respondents and respondents of Hausa ethnicity (Table 1).

**Table 1:** socio-demographic characteristics of respondents

Variables	Idi-Araba N (%)	Okokomaiko N (%)	Total N (%)	p-value
**Age group (years)**				NS
<25	49 (19.4)	30 (11.9)	79 (15.7)	
25-34	85 (33.7)	94 (37.3)	179 (35.5)	
35-44	58 (23.0)	66 (26.2)	124 (24.6)	
45-54	29 (11.5)	34 (13.5)	63 (12.5)	
55-64	19 (7.5)	20 (7.9)	39 (7.7)	
>64	12 (4.8)	8 (3.2)	20 (4.0)	
Median age, years (IQR)	32.0 (27-43)	35.0 (28-43.8)	34.0 (27-43)	NS
**Sex**				NS
Female	151 (59.9)	155 (61.5)	306 (60.7)	
Male	101 (40.1)	97 (38.5)	198 (39.3)	
**Marital status**				NS
Single	72 (28.6)	64 (25.4)	136 (27.0)	
Married	157 (62.3)	173 (68.7)	330 (65.5)	
Co-habiting	3 (1.2)	2 (0.8)	5 (1.0)	
Separated	7 (2.8)	2 (0.8)	9 (1.8)	
Divorced	2 (0.8)	2 (0.8)	4 (0.8)	
Widowed	11 (4.4)	9 (3.6)	20 (4.0)	
**Education**				NS
No formal	42 (16.7)	30 (11.9)	72 (14.3)	
Primary	50 (19.8)	67 (26.6)	117 (23.2)	
Secondary	104 (41.3)	114 (45.2)	218 (43.3)	
Post secondary	56 (22.2)	41 (16.3)	97 (19.2)	
**Religion**				NS
Christianity	118 (46.8)	138 (54.8)	256 (50.8)	
Islam	133 (52.8)	114 (45.2)	247 (49.0)	
Traditional	1 (0.4)	0 (0.0)	1 (0.2)	
**Ethnicity**				**<0.001**
Yoruba	134 (53.2)	158 (62.7)	292 (57.9)	
Hausa	66 (26.2)	17 (6.7)	83 (16.5)	
Igbo	33 (13.1)	64 (25.4)	97 (19.2)	
Others	19 (7.5)	13 (5.2)	32 (6.3)	
**Employment status**				**0.02**
Employed	186 (73.8)	207 (82.1)	393 (78.0)	
Unemployed	66 (26.2)	45 (17.9)	111 (22.0)	
**Occupation of the employed**				**<0.001**
Senior professional	3 (1.2)	3 (1.2)	6 (1.2)	
Intermediate professional	21 (8.3)	15 (6.0)	36 (7.1)	
Junior professional/skilled	43 (17.1)	45 (17.9)	88 (17.5)	
Semi-skilled	91 (36.1)	136 (54.0)	227 (45.0)	
Unskilled	28 (11.1)	8 (3.2)	36 (7.1)	
**Status of the unemployed**				NS
Housewife	28 (11.1)	18 (7.1)	46 (9.1)	
Student	25 (9.9)	20 (7.9)	45 (8.9)	
Apprentice	6 (2.4)	3 (1.2)	9 (1.8)	
Retired	7 (2.8)	4 (1.6)	11 (2.2)	

NS: not significant; IQR: inter quartile range

**Knowledge of TB:** Table 2 displays the correct responses to knowledge questions asked respondents. Three-quarters of respondents in the communities had heard about TB prior to the study. However, only 15.3% knew that TB is caused by a germ, 30.8% correctly responded that TB could be transmitted by air via a cough or sneeze and very few could properly identify the various signs and symptoms of TB (other than basic cough). Upon direct prompting, the least proportion (14.5%) of respondents knew that diabetics are at greater risk of TB followed by 34.3% who knew that people living with HIV were at greater risk. A quarter were aware of a health facility for TB diagnosis and treatment in their LGA while 11.1% and 9.1% knew of free diagnosis and free treatment for TB respectively. A significantly higher proportion of respondents in Idi-Araba (79.8%) had heard of TB than respondents in Okokomaiko (70.2%; p=0.01). Significantly higher proportions of respondents in Idi-Araba than in Okokomaiko mentioned cough and weight loss as symptoms of TB; knew that TB is transmissible; mentioned cross ventilation as a mode of prevention; knew that adults as well as children can be affected; mentioned that both genders can be affected and knew that people living with HIV are at greater risk of TB (p<0.05). Respondents reported the most common source of TB information to be family and friends (29.8%) followed by radio or television (26.2%) while only 10.1% got their information from health workers, 4% from newspapers or magazines, 3.4% from teachers, 0.8% from religious leaders, 0.6% from brochures or posters and 0.2% from other sources. More respondents in Idi-Araba (36.1%) got their TB information from family and friends while more respondents in Okokomaiko (26.2%) got theirs from radio or television; this difference was not statistically significant. The mean knowledge score was 9.8 ± 7.1, where 56.2% were designated as having good knowledge and 43.8% with relatively poor knowledge. The respondents in Idi-Araba had a significantly higher mean knowledge score than those in Okokomaiko (p=0.01).

**Table 2:** correct knowledge about general aspects of TB

Knowledge of TB	Idi-Araba N (%)	Okokomaiko N (%)	Total N (%)	p-value
**Heard of TB**	201 (79.8)	177 (70.2)	378 (75.0)	**0.01**
**Cause of TB a germ**	42 (16.7)	35 (13.9)	77 (15.3)	NS
**Signs and symptoms of TB**				
Cough	158 (62.7)	128 (50.8)	286 (56.7)	**0.01**
Coughing up sputum	9 (3.6)	23 (9.1)	32 (6.3)	**0.01**
Coughing up blood	60 (23.8)	81 (32.1)	141 (28.0)	**0.04**
Weight loss	91 (36.1)	60 (23.8)	151 (30.0)	**<0.01**
Fever	12 (4.8)	16 (6.3)	28 (5.6)	NS
Night sweats	4 (1.6)	2 (0.8)	6 (1.2)	NS
Chest pain	18 (7.1)	22 (8.7)	40 (7.9)	NS
Shortness of breath	13 (5.2)	12 (4.8)	25 (5.0)	NS
Loss of appetite	10 (4.0)	10 (4.0)	20 (4.0)	NS
Tiredness	8 (3.2)	13 (5.2)	21 (4.2)	NS
**Duration of cough regarded as suspicious for TB ≥2 weeks**	33 (13.1)	25 (9.9)	58 (11.5)	NS
**Transmission of TB**				
TB is transmissible	165 (65.5)	134 (53.2)	299 (59.3)	**0.01**
Transmitted through the air when a person with TB coughs/sneezes	81 (32.1)	74 (29.4)	155 (30.8)	NS
Through the air when a person with TB talks/sings	30 (11.9)	18 (7.1)	48 (9.5)	NS
**Mode of prevention of TB**				
Covering mouth and nose	59 (23.4)	49 (19.4)	108 (21.4)	NS
Avoid overcrowding	28 (11.1)	23 (9.1)	51 (10.1)	NS
Cross ventilation at home	15 (6.0)	5 (2.0)	20 (4.0)	**0.02**
Good nutrition	11 (4.4)	15 (6.0)	26 (5.2)	NS
BCG immunization in children	10 (4.0)	5 (2.0)	15 (3.0)	NS
**Age group and gender affected**				
Adults and children	173 (68.7)	150 (59.5)	323 (64.1)	**0.03**
Men and women	193 (76.6)	171 (67.9)	364 (72.2)	**0.03**
**Persons at greater risk of TB**				
Poor people	102 (40.5)	83 (32.9)	185 (36.7)	NS
Homeless people	117 (46.4)	98 (38.9)	215 (42.7)	NS
Alcoholics	115 (45.6)	109 (43.3)	224 (44.4)	NS
Drug users	119 (47.2)	100 (39.7)	219 (43.5)	NS
People living with HIV/AIDS	99 (39.3)	74 (29.4)	173 (34.3)	**0.02**
Prisoners	128 (50.8)	108 (42.9)	236 (46.8)	NS
Smokers	160 (63.5)	137 (54.4)	297 (58.9)	**0.04**
People who stay around smokers	128 (50.8)	88 (34.9)	216 (42.9)	**<0.001**
People who live with people with TB	144 (57.1)	116 (46.0)	260 (51.6)	**0.01**
People with diabetes	38 (15.1)	35 (13.9)	73 (14.5)	NS
**TB treatment**				
TB can be cured	177 (70.2)	149 (59.1)	326 (64.7)	0.01
Cured with specific drugs given at health facility	154 (61.1)	119 (47.2)	273 (54.2)	**<0.01**
DOTS	9 (3.6)	14 (5.6)	23 (4.6)	NS
Aware of health facility for TB diagnosis and treatment in LGA	76 (30.2)	50 (19.8)	126 (25.0)	0.01
Aware of free TB diagnosis in LGA	30 (11.9)	26 (10.3)	56 (11.1)	NS
Aware of free TB treatment in LGA	26 (10.3)	20 (7.9)	46 (9.1)	NS
**Overall knowledge**				
Good	155 (61.5)	128 (50.8)	283 (56.2)	**0.02**
Poor	97 (38.5)	124 (49.2)	221 (43.8)	
*Mean knowledge score, average*±SD	10.6 ± 7.0	9.0 ± 7.2	9.8 ± 7.1	**0.01**

NS: not significant; SD: standard deviation

**Attitudes towards TB:** of the respondents, 22% felt they could get TB - 66 from Idi-Araba and 44 from Okokomaiko. Eighty-eight (17.5%) respondents felt well-informed about TB; 53 from Idi-Araba and 35 from Okokomaiko. There were no statistically significant differences between the two groups. Data on attitude toward TB are shown in Table 3. Overall, a majority of patients understood TB to be a serious disease, including in Nigeria. A relatively small proportion (31.9%) of respondents were aware that HIV-infected patients should be concerned about TB. Most surprisingly, very few people (10.1%) perceived TB diagnosis and treatment to be free in Nigeria. Significantly higher proportions of respondents in Idi-Araba than in Okokomaiko correctly perceived TB to be a serious disease, considered TB to be a serious problem in Nigeria, would talk to a health personnel if they had TB, would go to a health facility with symptoms of TB, would support BCG immunization for children, and would like more information about TB (p<0.05). The mean attitude score was 5.3 ± 3.4, where 63.3% of respondents were designated as having a positive attitude and 36.7% with a negative attitude. The respondents in Idi-Araba had a significantly higher mean attitude score than those in Okokomaiko (p<0.01).

**Table 3:** attitudes towards TB

Questions on attitudes	Response	Idi-Araba N (%)	OkokomaikoN (%)	Total N (%)	p-value
In your opinion how serious a disease is TB?	Very serious	168 (66.7)	139 (55.2)	307 (60.9)	**0.01**
How serious a problem do you think TB is in Nigeria?	Very serious	146 (57.9)	119 (47.2)	265 (52.6)	**0.02**
Who would you talk to about your illness if you had TB?	Doctor or other medical worker	128 (50.8)	79 (31.3)	207 (41.1)	**<0.001**
What would you do if you thought you had symptoms of TB?	Go to health facility	191 (75.8)	163 (64.7)	354 (70.2)	**0.01**
If you had symptoms of TB, when would you go to health facility?	As soon as I realize symptoms might be TB	159 (63.1)	152 (60.3)	311 (61.7)	NS
Do you support BCG immunization for children?	Yes	198 (78.6)	174 (69.0)	372 (73.8)	**0.02**
How expensive do you think TB diagnosis and treatment is in Nigeria?	Free	29 (11.5)	22 (8.7)	51 (10.1)	NS
Which statement is closest to your feeling about people with TB disease?	“I feel compassion and desire to help”	152 (60.3)	147 (58.3)	299 (59.3)	NS
Do you think HIV+ people should be concerned about TB?	Yes	90 (35.7)	71 (28.2)	161 (31.9)	NS
Would you like to get more information about TB?	Yes	189 (75.0)	164 (65.1)	353 (70.0)	**0.02**
**Overall attitude**					
Positive		170 (67.5)	149 (59.1)	319 (63.3)	0.05
Negative		82 (32.5)	103 (40.9)	185 (36.7)	
*Mean attitude score, average* ± *SD*		5.8 ± 3.3	4.9 ± 3.5	5.3 ± 3.4	**<0.01**

NS: not significant; SD: standard deviation

**Preventive practices:** Table 4 shows the preventive practices of respondents. Over half of the respondents reported the practices of always covering mouth when coughing (65.1%), always covering nose/mouth when sneezing (57.7%) and always ensuring cross-ventilation at home (53%). Okokomaiko had significantly higher proportions of respondents that used hands and handkerchiefs to cover their mouths while Idi-Araba had a significantly higher proportion of respondents that always ensure cross ventilation at home (p<0.05). The mean practice score was 5.2 ± 1.5, where 48.8% of respondents were designated as engaging in good practices and 51.2% in poor practices. The respondents in Idi-Araba had a significantly higher mean practice score than those in Okokomaiko (p=0.04).

**Table 4:** respondents preventive practices

Practice	Idi-Araba N (%)	Okokomaiko N (%)	Total N (%)	p-value
**Covers mouth when coughing**				NS
Not at all	7 (2.8)	6 (2.4)	13 (2.6)	
Yes, sometimes	73 (29.0)	90 (35.7)	163 (32.3)	
Yes, always	172 (68.3)	156 (61.9)	328 (65.1)	
**Covers nose/mouth when sneezing**				**0.03**
Not at all	10 (4.0)	12 (4.8)	22 (4.4)	
Yes, sometimes	82 (32.5)	109 (43.3)	191 (37.9)	
Yes, always	160 (63.5)	131 (52.0)	291 (57.7)	
**Object(s) used to cover mouth**				
Hand	192 (76.2)	228 (90.5)	420 (83.3)	**<0.001**
Handkerchief	131 (52.0)	154 (61.1)	285 (56.5)	**0.04**
Crook of elbow	2 (0.8)	0 (0.0)	2 (0.4)	NS
Part of clothes	8 (3.2)	9 (3.6)	17 (3.4)	NS
**Ensures cross-ventilation in home**				**<0.001**
Not at all	27 (10.7)	12 (4.8)	39 (7.7)	
Yes, sometimes	62 (24.6)	136 (54.0)	198 (39.3)	
Yes, always	163 (64.7)	104 (41.3)	267 (53.0)	
**Overall practice**				
Good	135 (53.6)	111 (44.0)	246 (48.8)	**0.03**
Poor	117 (46.4)	141 (56.0)	258 (51.2)	
*Mean practice score*, *average* ±*SD*	5.3 ± 1.4	5.0 ± 1.5	5.2 ± 1.5	**0.04**

NS: not significant; SD: standard deviation

**Predictors of good knowledge, positive attitude and good practice:** bivariate analyses showed that there were statistically significant associations between age group, sex, education and level of knowledge; between education, occupation, knowledge of TB and attitude; between sex, occupation, knowledge of TB, attitude towards TB and practice. In multivariate analyses, predictors of good knowledge were increasing age, post secondary education and professional occupation. The predictors of positive attitude were post secondary education and good TB knowledge. Good knowledge was a predictor of good practice (Table 5).

**Table 5:** predictors of good knowledge, positive attitude and good practice

Characteristics	COR	AOR	95% CI	p-value
**Good knowledge^a^**				
Age	1.02	1.03	1.01-1.04	**<0.01**
Male Sex (vs. female)	1.45	1.20	0.82-1.76	NS
Post-Secondary Education (vs. <post-secondary)	2.09	2.08	1.27-3.42	**<0.01**
Professional Occupation (vs. non-professional)	1.84	1.65	1.06-2.56	**0.03**
**Positive attitude^b^**				
Male Sex (vs. female)	1.42	1.00	0.62-1.63	NS
Post-Secondary Education (vs. <post-secondary)	3.31	2.80	1.43-5.49	**<0.01**
Professional Occupation (vs. non-professional)	2.24	1.76	0.99-3.12	NS
Good Knowledge (vs. poor knowledge)	17.65	16.96	10.55-27.27	**<0.001**
**Good practice^c^**				
Male Sex (vs. female)	1.46	1.35	0.93-1.96	NS
Professional Occupation (vs. non-professional)	1.37	1.14	0.75-1.74	NS
Good Knowledge (vs. poor knowledge)	2.04	1.79	1.15-2.79	**0.01**
Positive Attitude (vs. negative attitude)	1.76	1.19	0.75-1.88	NS

COR: crude odds ratio; AOR: adjusted odds ratio; NS: not significant

Reference categories: ^a^ Poor knowledge, ^b^ Negative attitude, ^c^ Poor practice

## Discussion

To date, few studies have been published that assessed community KAP regarding TB in Nigeria and fewer still have been conducted in urban slum communities, despite the high risk for TB in these settings [12]. The two communities included in this study were comparable in most of the socio-demographic variables examined. A large majority of respondents had heard of TB prior to the survey, similar to the reported rate of 74.7% from the Nigerian 2008 Demographic and Health Survey [13]. However, a more recent study demonstrated higher TB awareness (97.3%) within a rural community in South-East Nigeria possibly because of TB workshops, seminars and public lectures given within that community coupled with health talks on TB routinely given at primary health care centers [10]. Very few respondents got their TB information from health workers, underscoring the poor penetration of TB control activities within communities. TB education strategies proposed by the government include decentralization of TB care and control services into the community [14]; additionally, we recommend closer interactions between health workers and communities to ensure that more factual information is being passed on than would received from the more common sources of family, friends and radio or television. The mean knowledge score of respondents in this study was low. Using the mean score as a cut-off, just over half of respondents had good knowledge. Particularly worrisome were the very low proportions that knew TB was caused by a germ; that mentioned common signs of TB such as coughing up sputum, fever, night sweats, chest pain and shortness of breath and that knew duration of cough regarded as suspicious for TB. Similarly, there was a poor understanding of the cause of TB and its symptoms within a rural community in South-East Nigeria [10] and among newly diagnosed TB patients in South-West Nigeria [15]. Wrong beliefs about the cause of TB are thought to be promoters of TB stigma and delayed health-seeking behaviors in underserved communities [16]. Alarmingly, less than two-thirds of the respondents knew about TB transmission through the air and very few knew of the various means of preventing TB, availability of free diagnosis and treatment for TB, or of facilities for diagnosis and treatment. Poor knowledge in these areas means community members are less likely to take preventive measures against TB and more likely to delay or avoid seeking care even if symptoms arise [16, 17]. Indeed, in this study, good knowledge of TB was found to be a predictor of both positive attitude and good preventive practices against TB. Health education interventions are urgently needed and should stress to community residents that cough of more than two weeks can be investigated free of charge in neighboring health facilities.

Another area of note was the inability of most respondents to recognize the risk for TB among people living with HIV. This was better understood in a national household survey in South Africa [18]. Lack of information on the association between TB and HIV has been shown to negatively influence HIV testing among TB patients in Uganda [19] and South Africa [20]. This association is important information to be circulated within communities at high-risk for TB. Despite the high burden of TB in Nigeria, just about half of respondents in both slums perceived it to be a serious problem in Nigeria and less than three-quarters perceived it to be a very serious disease. In a different study, 91.2% of patients in Southeast Nigeria that had TB perceived the disease to be serious, possibly because they received more TB information at the health facility after diagnosis [21]. TB sensitization and control programs should emphasize the seriousness of the disease as this would ensure that community members seek care if they develop signs and symptoms of TB. Overall, most of the respondents in both communities had positive attitude towards TB particularly in their willingness to go to the health facility if they thought they had TB, in their support of BCG immunization and their willingness to get more information on TB. Public health interventions can leverage on this by using multiple methods to disseminate TB information and ensuring linkages with care for diagnosis and treatment of TB. Close to half of the respondents in both communities had good preventive practices against TB. Their main area of deficiency was in the use of their hands to cover their mouths when coughing. Similarly, high-risk respiratory hygiene practices were also observed in densely populated communities in Bangladesh [22]. We recommend demonstrations of appropriate respiratory hygiene techniques during public health interventions. Upon comparison, higher proportions of respondents from Idi-Araba had good knowledge, positive attitudes and good preventive practices than Okokomaiko. A possible reason for this could be because Idi-Araba respondents live in close proximity to a government tertiary facility, which carries out outreach programmes into the community from time to time and respondents could have possibly been sensitized about TB prior to the survey. In the final analysis, having post secondary education was a predictor of both good knowledge and positive attitude towards TB. Higher education has consistently been shown to positively influence knowledge of TB in other African studies in South Africa [18] and Ethiopia [23]. This further underscores the need to improve the educational level within communities so as to enhance their health status generally as education is a fundamental social determinant of health [24].

## Conclusion

The identified gaps in TB knowledge, attitudes and practice in this study underscore the need to improve the education about TB in underserved communities. The findings may be of benefit in the planning of public health interventions to improve KAP scores, which will ultimately lead to higher rates of TB detection and treatment.

### What is known about this topic

- Similar studies in Nigeria have demonstrated poor knowledge of TB within communities;
- Attitudes towards TB have also been well studied in previous studies.

### What this study adds

- This study explores not only knowledge and attitudes towards TB but also the preventive practices of community members;
- This study also contributes to the body of TB research focused on vulnerable communities such as urban slums, which has not been emphasized in previous literature from Nigeria;
- This study provides useful information about the predictors of good knowledge, positive attitudes and good practices.

## Competing interests

The authors declare no competing interests.
